# Mitochondria, a Missing Link in COVID-19 Heart Failure and Arrest?

**DOI:** 10.3389/fcvm.2021.830024

**Published:** 2022-01-17

**Authors:** Ralph Ryback, Alfonso Eirin

**Affiliations:** ^1^Mindful Health Foundation, Naples, FL, United States; ^2^Department of Internal Medicine, Division of Nephrology and Hypertension, Mayo Clinic, Rochester, MN, United States

**Keywords:** COVID-19, mitochondria, heart failure, cardiovascular disease, ATP

Over the last 2 years, we have all been trying to understand the interrelationships of COVID-19's numerous symptoms, clinical risk factors, and lethality. Autopsy cases of patients with COVID-19 revealed that the virus was present in the heart of more than 60% of patients, associated with evidence of active viral replication, suggesting direct viral cardiac infection (1). Contrarily, a recent study reported that the virus was detected in the heart of only one out of 30 patients who died after a prolonged hospital stay due to Sars-Cov-2 infection., associated with modest histological alterations (2). Macroscopic, histological, and immunohistochemical analysis revealed modest cardiac histological alterations, underscoring the lack of evidence to establish the contribution of a direct effect of SARS-CoV-2 on cardiac lesions. Mehra and Ruschitzka (3) noted in the elderly, especially with cardiovascular disease, mortality was associated with a very significant elevation of natriuretic peptides (NPs) with death attributed to cardiac failure and arrest in almost 25% of cases. They wondered whether cardiac inflammation or dysfunction suggested by elevated NP's might play a role in the respiratory hypoxic failure observed in COVID-19. NPs, hormones secreted from the heart, have many functions including promoting Na^+^, excretion by the kidney. In addition, NPs are involved in important mitochondrial mediated processes including Ca^2^+ signaling, apoptosis, reactive oxygen species production, biogenesis, and fat oxidation, etc. (4). Cardiac followed by kidney cells have the highest mitochondrial content (high ATP energy needs) and thus their function is directly dependent on mitochondrial health (4). However, cardiac mitochondrial function extends well-beyond energy production and includes modulation of numerous cellular signaling pathways at molecular and biochemical levels (5). Thus, mitochondrial damage has a tremendous impact on overall cardiomyocyte function.

Evidence reveals the virus localizes to mitochondria which it attacks and disrupts (Figure 1), thereby taking energy away from the cells' battle with the virus including autophagy (6, 7). In this process, SARS-CoV-2 manipulates mitochondrial function by angiotensin-converting enzyme 2 (ACE2) regulation and open-reading frames (ORFs) to evade host cell immunity and facilitate virus replication. The virus-encoded protein Orf-96 localizes to mitochondria and triggers degradation of mitochondria-related genes, including DRP1, MAVS, TRAF3, and TRAF6 (8). ORFs, such as ORF3a, can target the mitochondrial deubiquitinase USP30, altering mitochondrial homeostasis (biogenesis, fusion, fission, and mitophagy) and function (9). Furthermore, the 3a protein of the virus promotes mitochondrial apoptosis (10). In cellular homeostasis, there is a balance between the BCL-2 family protein, which are neuroprotective and Bax proteins which can be transformed to set off a cell-death cascade. This can occur in response to extracellular stimulation by stress, viral infection, excessive immune cytokines secretions, etc. Bax exist in a relatively stable molecular form, but with viral infection it changes form and moves to the outer membrane of the mitochondria where it inserts itself, causing the release of the cytochrome c, initiating apoptosis (10) with the release of its DNA. In addition, the 3a protein promotes activation of truncated Bid (tBid) which form pores in the mitochondria, favoring the release of apoptogenic factors. The unique DNA of the degraded mitochondria is then released into the blood whose presence at high levels has now been reported (11) to predict poor COVID-19 outcomes.

**Figure 1:**
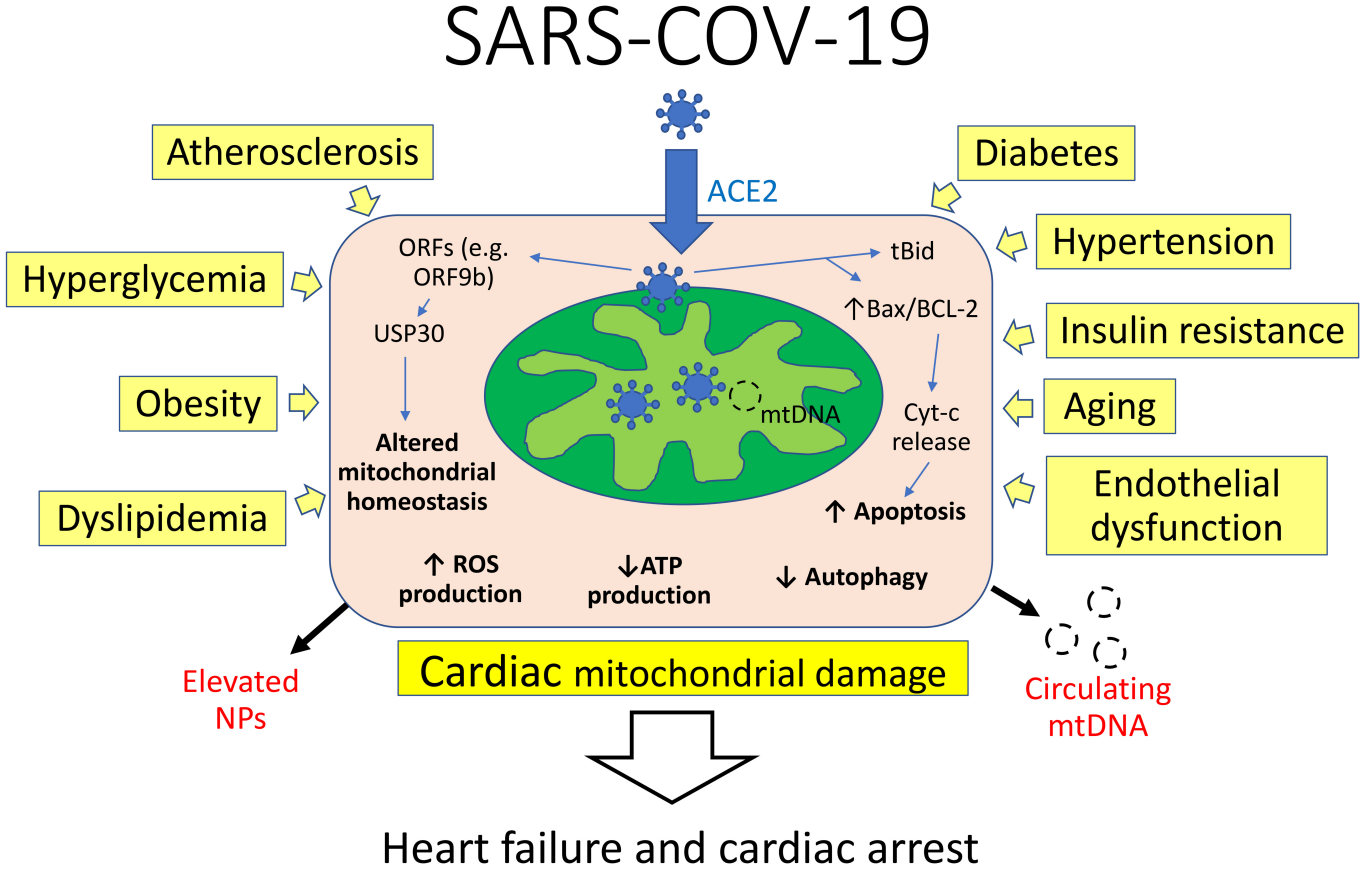
Interplay of mechanisms of cardiac mitochondrial damage in COVID-19. Viral RNA and protein localize to mitochondria and manipulate their function by angiotensin-converting enzyme 2 (ACE2) regulation and open-reading frames (ORFs) to evade host cell immunity and facilitate virus replication. ORFs can target the mitochondrial deubiquitinase USP30, altering mitochondrial homeostasis and function. The virus promotes activation of truncated Bid (tBid) and alters Bax/BCL-2 ratio, favoring the release of apoptogenic factors. Concomitantly, several cardiovascular risk factors can compromise mitochondrial integrity and function by decreasing ATP levels and increasing reactive oxygen species (ROS) production. This in turn leads to cardiac mitochondrial damage and heart failure, associated with elevation of natriuretic peptides (NPs) and circulating mitochondrial DNA (mtDNA).

Importantly, pre-existent mitochondrial damage might exacerbate cardiomyocyte injury and dysfunction. It seems pertinent that all of the clinical risk factors (atherosclerosis, age, obesity, hypertension, and other conditions such as endothelial dysfunction) share impaired mitochondrial respiration, or a decrease in its ability to produce ATP. Hypertension, one of the main risk factors for SARS-COV and SARS-COV-19, is “prominently associated with the loss of cardiolipin” (12), a phospholipid uniquely found in the inner mitochondrial membrane and necessary for its proper formation and function. Furthermore, cardiolipin regulates mitochondrial dynamics and prevents the formation and opening of the mitochondrial permeability transition pore (mPTP), and release of cytochrome C to the cytosol. Hypertension is part of a process whereby a hole is opened in the “armored nuclear power plant” of the mitochondria that can be pierced and destroyed by SARS-COV-19.

Similarly, insulin resistance in skeletal muscle, a major hallmark of type 2 diabetes and obesity, has been linked to decreased muscle mitochondria reproduction and dysfunction (13). Endothelial cells exposed to high glucose concentrations exhibit augmented mitochondrial superoxide generation, which damages lipids, proteins, and mtDNA, and contributes to cellular oxidative stress (14). The uncoupled mitochondrial state is necessary for ATP synthesis. Excessive mitochondrial coupling is a central expression of “dysfunction in obesity that may contribute to the development of metabolic pathologies such as insulin resistance and diabetes” (15). Cardiac mitochondria also deteriorate with age, losing respiratory activity, accumulating damage to their DNA (mtDNA), and producing excessive amounts of reactive oxygen species (ROS), ultimately increasing susceptibility to infections (16). Therefore, results from these studies are consistent with established mitochondrial injury that may aggravate cardiac damage and accelerate COVID-19-related mortality rates in patients with cardiovascular risk factors.

Recent evidence suggests that cardiac troponin I (cTnI), an important structural protein implicated in contraction and relaxation of cardiomyocytes, is a critical biomarker of myocardial injury in COVID-19 and is directly related to survival (17). Interestingly, mitochondrial structure and function are significantly impaired in cardiomyocytes with mutated cTnI, suggesting an important role of this protein in maintaining the structural and functional integrity of myocardial mitochondria (18). Thus, monitoring cTnI may be useful to assess cardiac mitochondrial damage and disease progression in patients with COVID-19.

Beyond mediating damage of infected cardiac cells, mitochondria are emerging as critical components of the innate immune response. It has been shown that the ATP needed for purinergic signaling (e.g., adenosine and ATP), T-cell regulation, and initial activation of neutrophils comes from mitochondria. ATP production and mitochondrial Ca^2+^ buffering are needed for antigen presentation and processing, and ROS are a part of the signaling pathway that activates inflammatory proteins (19). As mediators of immunity, mitochondria are consequently targeted by several viruses, including the SARS-CoV-2 virus. As noted, Orf-96 localizes on the mitochondrial membrane and suppresses type 1 interferon responses (20). Immune cells (and all cells) cannot function without their multiple healthy mitochondria. With aging, immune T cells don't respond as well to pathogens or vaccines as T cells' mitochondria begin to malfunction. This is reflected in the age related cognitive, cardiovascular, physical, metabolic, etc. changes, experienced and observed. Nevertheless, poor T cell response might not only be the result of aging but may be part of the cause of aging by releasing excessive inflammatory cytokines (the cytokine storm). When T cell mitochondria had been genetically modified (TFAM deficiency) to be energy production inefficient, it forced T cells from ATP into a less efficient mode of energy production. These mice rapidly aged with deterioration in their functions noted previously. “T cell metabolic failure induces the accumulation of circulating cytokines, characteristic of aging (‘inflammaging'). This cytokine storm itself acts as a systemic inducer of senescence” (21).

A major immune defense against viral infection necessary for cellular viability, is autophagy. It delivers viral proteins and viruses to lysosomes for degradation. However, lysosomes are impaired by the loss of mitochondrial function (22) such as in SARS-COV, and COV-19 related impairments. “Inflammaging” and decreased autophagy accelerate the metabolic compromised state of people with known risk factors. It is no surprise that our young are more resilient since they usually generate sufficient ATP. However, when elevated blood mtDNA is found even in seemingly younger healthier patients and others, it reflects severe complications that can lead to ICU care and even death (11). All of the 97 adult subjects had COVID-19, but those that died had higher cell free plasma levels of mtDNA and fragments derived from mitochondrial encoded gene cytochrome B (MT-CYTB). MT-CYTB levels were highly correlated with plasma SC5b-9, which is “a marker of complement activation and suggests the formation of a membrane attack complex” (11).

Many questions remain unanswered including, is the appearance of mtDNA just part of an “over exuberant innate immune response?” (11). Would viral infection trigger cellular necrosis if their mitochondria remained more intact? Does viral induced mitochondrial dysfunction underlie the myocardial injury observed? Does the appearance of plasma mtDNA and MT-CYTB fragments portend a possible cascade of negative immunologic responses? Are the extreme elevations in NPs observed in part a hormonal response to protect and stabilize failing cardiac mitochondrial respiration and/or an expression thereof? Given the importance of mitochondria in kidney function, could their failure be expressed as excess deaths from end-stage kidney failure early in the pandemic? (23). Could finding ways to protect cardiac mitochondrial function open a whole new field of prevention or even treatment?

Studies have demonstrated that normalizing tubular cell mitochondrial function and energy balance could be a preventative strategy in kidney disease (24). Moreover, targeting the regulation of mitochondrial biogenesis and/or correcting abnormal electron chain function, can improve renal disease outcome. Could acute IV infusion of beta-hydroxybutyrate, the body's primary ketone body, improve cardiac mitochondrial respiration? This is supported by studies in healthy, and heart failure patients showing improved hemodynamic and cardiac output (25). Undoubtedly, additional studies are needed to establish the exact role of cardiac mitochondrial damage in the setting of COVID-19 and heart failure.

## Author Contributions

RR and AE conceived the manuscript, revised the drafts, and approved the submitted version. Both authors contributed to the article and approved the submitted version.

## Funding

Partly supported by the NIH grant DK129240.

## Conflict of Interest

The authors declare that the research was conducted in the absence of any commercial or financial relationships that could be construed as a potential conflict of interest.

## Publisher's Note

All claims expressed in this article are solely those of the authors and do not necessarily represent those of their affiliated organizations, or those of the publisher, the editors and the reviewers. Any product that may be evaluated in this article, or claim that may be made by its manufacturer, is not guaranteed or endorsed by the publisher.
